# Ascitic Shear Stress Activates GPCRs and Downregulates Mucin 15 to Promote Ovarian Cancer Malignancy

**DOI:** 10.21203/rs.3.rs-5160301/v1

**Published:** 2024-10-15

**Authors:** Eric N. Horst, Liam R. Cotter, Mia Bonini, Caymen M. Novak, Nina M. Treacher, Yeye Zhang, Zoe F. Jackson, Ishwarya V. Narayanan, Zachary S. Fischer, Alec R. Sunshine, Zequan Lin, Linh A. Tran, Mats Ljungman, Katherine E. Maturen, Analisa DiFeo, David A. Nordsletten, Geeta Mehta

**Affiliations:** Department of Biomedical Engineering, Michigan Medicine, College of Engineering, University of Michigan, Ann Arbor, 48109; Department of Materials Science and Engineering, College of Engineering, University of Michigan, Ann Arbor, MI, 48109; Department of Biomedical Engineering, Michigan Medicine, College of Engineering, University of Michigan, Ann Arbor, 48109; Department of Mechanical Engineering, College of Engineering, University of Michigan, Dearborn, MI, 48128; Department of Biomedical Engineering, Michigan Medicine, College of Engineering, University of Michigan, Ann Arbor, 48109; Department of Biomedical Engineering, Michigan Medicine, College of Engineering, University of Michigan, Ann Arbor, 48109; Department of Biomedical Engineering, Michigan Medicine, College of Engineering, University of Michigan, Ann Arbor, 48109; Department of Radiation Oncology, Michigan Medicine, University of Michigan, Ann Arbor, MI, 48109; Department of Biomedical Engineering, Michigan Medicine, College of Engineering, University of Michigan, Ann Arbor, 48109; Department of Biomedical Engineering, Michigan Medicine, College of Engineering, University of Michigan, Ann Arbor, 48109; Department of Biomedical Engineering, Michigan Medicine, College of Engineering, University of Michigan, Ann Arbor, 48109; Department of Materials Science and Engineering, College of Engineering, University of Michigan, Ann Arbor, MI, 48109; Rogel Cancer Center, Michigan Medicine, University of Michigan, Ann Arbor, MI, 48109; Department of Radiation Oncology, Michigan Medicine, University of Michigan, Ann Arbor, MI, 48109; Department of Environmental Health Sciences, School of Public Health, University of Michigan, Ann Arbor, MI, 48109; Center for RNA Biomedicine, University of Michigan, Ann Arbor, MI, 48109; Department of Radiology, Michigan Medicine, University of Michigan, Ann Arbor, MI, 48109; Department of Obstetrics and Gynecology, Michigan Medicine, University of Michigan, Ann Arbor, MI, 48109; Department of Pathology, Michigan Medicine, University of Michigan, Ann Arbor, MI, 48109; Rogel Cancer Center, Michigan Medicine, University of Michigan, Ann Arbor, MI, 48109; Department of Obstetrics and Gynecology, Michigan Medicine, University of Michigan, Ann Arbor, MI, 48109; Cancer Biology Graduate Program, Michigan Medicine, University of Michigan, Ann Arbor, MI, 48109; Department of Biomedical Engineering, Michigan Medicine, College of Engineering, University of Michigan, Ann Arbor, 48109; Department of Cardiac Surgery, Michigan Medicine, University of Michigan, Ann Arbor, MI, 48109; Department of Biomedical Engineering, Michigan Medicine, College of Engineering, University of Michigan, Ann Arbor, 48109; Department of Materials Science and Engineering, College of Engineering, University of Michigan, Ann Arbor, MI, 48109; Rogel Cancer Center, Michigan Medicine, University of Michigan, Ann Arbor, MI, 48109; Macromolecular Science and Engineering, College of Engineering, University of Michigan, Ann Arbor, 48109

**Keywords:** mechanotransduction, ascitic currents, transcoelomic metastasis, fluid shear stress, high grade serous ovarian cancer, G-protein coupled receptors (GPCR), mucin 15 (MUC15), glycocalyx, NF-κB, epithelial to mesenchymal transition (EMT), PLCβ

## Abstract

The accumulation of ascites in patients with ovarian cancer increases their risk of transcoelomic metastasis. Although common routes of peritoneal dissemination are known to follow distinct paths of circulating ascites, the mechanisms that initiate these currents and subsequent fluid shear stresses are not well understood. Here we developed a patient-based, boundary driven computational fluid dynamics model to predict an upper range of fluid shear stress generated by the accumulation of ascites. We show that ovarian cancer cells exposed to ascitic shear stresses display heightened G protein-coupled receptor mechanosignaling and the induction of an epithelial to mesenchymal-like transition through p38α mitogen-activated protein kinase and mucin 15 modulation. An emergent immunomodulatory secretome and endoplasmic reticulum stress activation is also present in shear stimulated cancer cells, positioning elevated shear stress as a protumoural signal. Together, these findings suggest maintenance strategies for overcoming mechanotransduction mediated metastasis within the peritoneal cavity.

## Introduction

A central challenge in the management of high grade serous ovarian cancer (HGSC) is its capacity to undergo rapid and widespread transcoelomic metastasis. This process, wherein disseminating cancer cells peregrinate through ascitic currents to secondary sites within the peritoneum, relies on ascites rather than plasma as the medium of transport^1–3^. Extensive transcoelomic metastasis and advanced staging (i.e., stages III and IV) are present in 70% of patients at the time of diagnosis^1^. Average ascites volume increases significantly with the stage of disease, from 300 mL at stage I to 2460 and 2810 mL at stages III and IV respectively^4^. Though ascites volume is closely correlated with advanced disease stage and poor prognosis^5,6^, its role in the initiation of ascitic currents and the mechanotransduction of the corresponding ascitic fluid shear stress (FSS) by tumour cells remains unclear^7^.

Interstitial FSS is ubiquitous in solid tumours (FSS ≤ 1 dyne/cm^22^), where it enhances both cancer stemness and metastatic potential^8,9^. FSS, first sensed by the glycocalyx^10^, is amplified at lipid rafts and caveolae, where membrane proteins such as caveolin 1^11^, integrins^12^, tyrosine kinase receptors^13^, G protein-coupled receptors (GPCR)^14^, ion channels^15^, and downstream signaling like PI3K-Akt are activated. It is unknown however, which of these processes is conserved in FSS-led mechanotransduction in ovarian cancer^7^. Previous studies have utilised estimates of FSS in HGSC^16,17^. For example, oncotic gradients across the peritoneum^16^ (FSS ≤ 0.1 dyne/cm^2^) activate the drug efflux mechanism miR-199a-3p-PI3K/Akt-ABCG2/P-gp^9^ and drive epithelial to mesenchymal transition (EMT) through E-cadherin loss and epidermal growth factor receptor (EGFR) modulation^18^. Gastrointestinal peristalsis^17^ is also hypothesised to transmit wall shear stress to the peritoneal cavity (FSS ≤ 2.7 dyne/cm^22^), resulting in the recruitment of cytoskeletal stress fibers^19^ and *cooling* of the immune response through NF-κB activation^20^. Although foundational, these studies are limited by a lack of patient informed pathophysiology which neglects the mechanisms that initiate ascites fluid motion and thereby underestimate FSS within the peritoneal cavity.

Ascites establishes an active resistance to diaphragmatic excursion, as evidenced by a 92% increase in patients’ elastic work of breathing^21,22^. Increased muscle contraction against the peritoneum transfers contractile forces to the cavity producing subdiaphragmatic pressures. This cyclic loading displaces ascites fluid resulting in the formation of ascitic (or cephalic) currents^23,24^. We therefore posit that ascitic currents generate from cyclic diaphragmatic excursion and that FSS experienced by the primary ovarian tumour is greater than formerly appreciated^7^.

In order to establish biologically relevant ascites currents, we utilised computational modeling to estimate an upper range of FSS from the anatomy of an HGSC patient. Previously we have used 3D computational fluid dynamics (CFD) in conjunction with medical images to simulate biofluid flows^25,26^. Using a similar approach, we engineered an in silico model from clinically available segmented 3D computed tomography (CT) scans and prescribed deformation driven by diaphragm movement to inform ascitic current formation within the peritoneum. Next, we embedded HGSC cell lines within our tumour mimetic semi-interpenetrating network hydrogel (IPN)^27^, and exposed them to kinematic model educated ascitic FSS within a pulsatile perfusion bioreactor^28^. We found a unique shear conditioned mesenchymal-like phenotype which was dependent on GPCR and mucin 15 (MUC15) FSS sensing and downstream p38α MAPK and NF-κB signaling. Ascitic FSS was also found to support a protumoural and immunomodulatory secretome, with a stimulated cytokine production mirroring that found within patient ascites^1^. Together, by mechanistic investigation of ascites pathophysiology, MUC15, and GPCR signaling, our study provides a fresh perspective on ascitic currents, the roles of the glycocalyx in cancer progression, and modalities of epithelial cell mechanosensing within high shear environments.

## Results

### Formation of ascitic currents and resultant FSS

**Figure 1:**
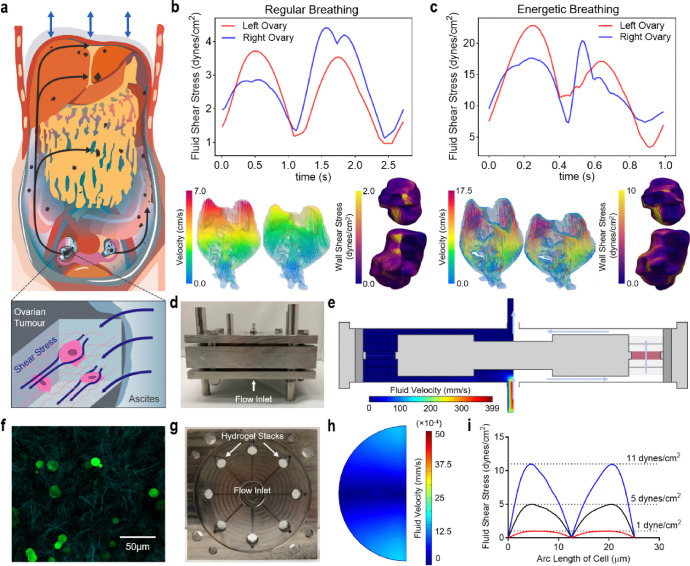
Ascitic currents impart elevated FSS on ovarian tumours. **a**, Ascitic fluid motion from diaphragmatic excursion creates elevated FSS and promotes ovarian cancer metastasis. **b,c,** Plot of the maximum fluid shear stress on left and right ovary for 1 breathing cycle (top; dynes/cm^2^), velocity fields of the ascites fluid at the start and middle of the breathing cycle (bottom left; cm/s), and FSS plotted on the ovaries at t = 1.5 s and t = 0.3 s for the case of regular (**b**) and energetic breathing (**c**), respectively. **d**, Assembled FSS bioreactor. e, Fluid velocity profiles in a FSS bioreactor radial section (mm/s). **f**, Fluorescent and second harmonic generation imaging of GFP-expressing OVCAR3 suspended in an agarose/collagen type I interpenetrating network hydrogel. **g**, Top-down view of the FSS bioreactor showing the radial chambers for cell-laden hydrogel samples. **h**, Cross-section of fluid velocity through the center of an agarose/collagen IPN hydrogel. **i**, FSS over the arclength of an idealised cell (dynes/cm^2^).

Growing oncotic pressures drive exudative ascites to accumulate within the peritoneal cavity, in turn promoting HGSC dissemination, resistance to anoikis, and resulting transcoelomic metastasis^1^. During this dissemination, ascites spreads tumour cells throughout the abdominal viscera, traversing distinct compartments (e.g. right and left paracolic gutters, subphrenic space, and Morrison’s pouch) to reach frequent sites of metastasis including the lung, liver, and omentum^23^. Diaphragmatic excursion during breathing sustains cyclic subdiaphragmatic pressures which physically circulate ascites through these channels, exposing the primary ovarian tumour to elevated fluid motion and resultant FSS (Fig. 1a).

To investigate the formation of ascitic currents and provide an upper range of FSS within the peritoneal cavity, we utilised patient-specific CFD modeling. CFD allows for the prediction of biofluid motion and complex metrics (e.g. wall-shear stress) which are not measurable using standard clinical methods^25,29^. Ascitic fluid flow was modeled for normal respiration (regular breathing) and under active conditions (energetic breathing) (**Supplementary Video 1–4** and **Extended Data** Fig. 1a). Results from the regular breathing model show a peak velocity of 8.21 cm/s and a mean velocity of 1.66 cm/s in the ascites (Fig. 1b). These flow patterns result in an average FSS of 0.27 and 0.28 dynes/cm^2^ and a peak FSS of 3.71 and 4.41 dynes/cm^2^ on the left and right ovary, respectively. For the active model, the respective mean and peak velocities in the ascites are 5.92 and 26.1 cm/s. This results in an average FSS of 1.33 and 1.51 dynes/cm^2^ and a peak FSS of 22.8 and 20.5 dynes/cm^2^ on the left and right ovary, respectively (Fig. 1c). To investigate the effects of ascitic FSS in vitro, we milled a perfusion shear stress bioreactor to apply derived FSS values to HGSC cells (Fig. 1d and **Extended Data** Fig. 1b-d). Fluid velocity profiles within the bioreactor were calculated using a finite element model as previously established^28^ (COMSOL Multiphysics; Fig. 1e and **Extended Data** Fig. 1e). Briefly, fluid velocity in the cell-laden tumour-like hydrogel compartment was averaged for use in a secondary model of an idealised cell.

Ovarian tumour extracellular matrix (ECM) is heterogeneous, stiff (5–35 kPa), and comprised of high molecular weight molecules (e.g. glycosaminoglycans and polysaccharides) with tensile and interpenetrating bioactive fibers (e.g. collagen)^30^. To model this ovarian tumour specific ECM, we previously characterised a semi-IPN hydrogel comprising 3% (w/v) agarose with 1 mg/mL collagen type I (Fig. 1f and **Supplementary Video 5**) for use in mechanical stress bioreactors^27^. Hydrogels, each containing 10 million cells, were injected into the 8 radial chambers of the bioreactor for FSS stimulation (Fig. 1g). We chose three values of FSS to represent interstitial fluid flow (1 dyne/cm^2^) and both the average and elevated ascitic flow from diaphragmatic excursion (5 and 11 dynes/cm^2^, respectively). The associated input velocities (Fig. 1h,i) were generated by finite element modeling of a theoretical HGSC cell in an open flow channel^28^.

### Establishment of a shear mediated phenotype in HGSC cells

**Figure 2:**
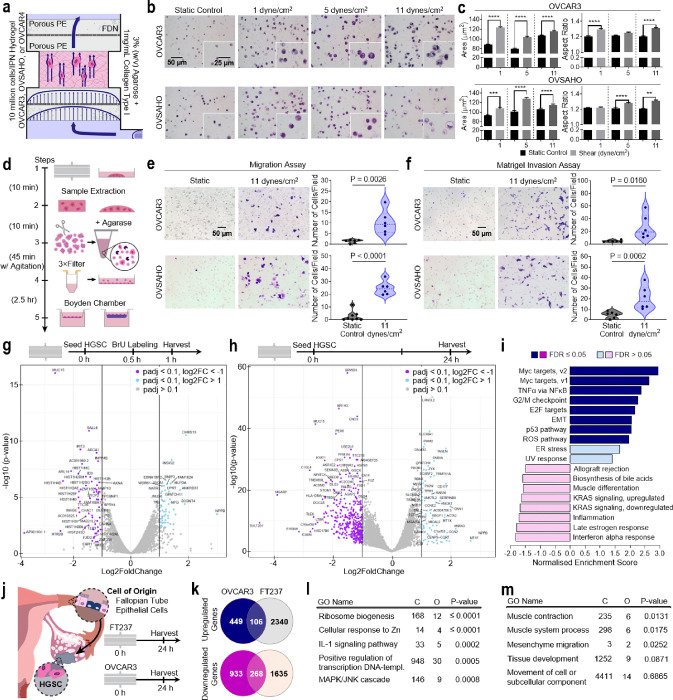
FSS alters the phenotype of ovarian cancer cells. **a,** Schematic of in vitro FSS stimulation of ovarian cancer cells. 10 million cells were exposed to FSS in an agarose/collagen type I hydrogel. Porous polyethylene (PE) disks and stainless-steel fluid distribution nets (FDNs) create a uniform, laminar flow distribution. Arrows indicate the direction of fluid flow**. b,c**, Hematoxylin and eosin stains (**b**) of static or sheared OVCAR3 (top) and OVSAHO (bottom) at increasing FSS magnitudes (1 dynes/cm^2^, 5 dynes/cm^2^, 11 dynes/cm^2^; 24 hr) and the associated quantification of cell area and aspect ratio (**c**; n = 3). **d**, Experimental timeline for static and FSS treated cell recovery for use in Boyden chamber migration and Matrigel invasion assays. **e**, Boyden chamber migration assays using static and sheared OVCAR3 (top) and OVSAHO (bottom) cells (11 dynes/cm2, 24 hr). Quantification of the number of migrated cells per field of view (FOV). f, Boyden chamber Matrigel invasion assays using static and sheared OVCAR3 (top) and OVSAHO (bottom) cells (11 dynes/cm^2^, 24 hr). Quantification of the number of invaded cells per FOV. **g**, Bru-seq volcano plot of differential nascent RNA expression upon shearing OVCAR3 cells (11 dynes/cm2, 1 hr). **h**, Bulk RNA-seq volcano plot of differential mRNA expression after shearing OVCAR3 cells (11 dynes/cm^2^, 24 hr). **i**, GSEA of RNA-seq data showing gene sets upregulated and downregulated in OVCAR3 following a 24 hr exposure to 11 dynes/cm^2^. Gene sets in the Hallmark pathways are shown and the data is expressed as normalised enrichment scores (NES). **j**, RNA-seq was performed on static and sheared OVCAR3 and FT237 cells (11 dynes/cm^2^, 24hr). **k**, Comparison of the shear-induced differential gene expression between OVCAR3 and FT237. **l,m**, Network Topology Analysis (NTA) of RNA-seq data showing uniquely upregulated genes in sheared OVCAR3 when compared to sheared FT237, gene sets in TCGA RNAseq TGCT (**I**) and ACC (**m**) datasets are shown. Significance evaluated by Student’s t-test. **P ≤ 0.01, ***P ≤ 0.001, ****P ≤ 0.0001.

To probe the impact of ascitic FSS on HGSC, we applied FSS (1, 5, and 11 dynes/cm^2^) to OVCAR3 and OVSAHO representing HGSC immortalised cell lines within tumour mimetic agarose/collagen type I IPN hydrogels (Fig. 2a). After exposure to interstitial flow (1 dyne/cm^2^; 24 hr), embedded cancer cells increased in cellular area and elongated (Fig. 2b,c and **Extended Data** Fig. 2a), as found previously^19^. Next, we exposed HGSC cells to elevated FSS representing ascitic circulation (5 and 11 dynes/cm^2^; 24 hr) and determined that the morphological changes were conserved in the higher shear regime. Cellular proliferative marker Ki67 also increased in both OVCAR3 and OVSAHO at the upper level of FSS (**Extended Data** Fig. 2b,c). As FSS stimulation has been shown to enhance migratory potential in ovarian cancer, we used a prior live-cell recovery method to retrieve stimulated and static-control cells from agarose/collagen IPN hydrogels (Fig. 2d)^18,19,27^. Boyden chamber assays seeded with recovered cells showed significantly enhanced migration (P = 0.0026 and P < 0.0001) and invasion (P = 0.016, and P = 0.0062; OVCAR3 and OVSAHO) after FSS exposure (Fig. 2e,f).

To assess the mechanisms underlying this shear-induced phenotype we performed a series of bulk mRNA analysis. First, to probe early molecular responses to mechanosensing, we recovered nascent mRNA (Bru-seq) at the onset of FSS (0.5–1 hr). Static controls and sheared samples clustered tightly, with a 66% variance and a 27% variance in the primary and secondary vectors respectively by principal component analysis (PCA; **Extended Data** Fig. 2d). Intriguingly, there were no clear alterations to the transcription of genes expected in the process of mechanotransduction (*YAP/TAZ, PIEZO1/2, Rho/ROCK*, etc.; Fig. 2g)^31^. However, transcription of the transmembrane glycoprotein, MUC15, whose loss is observed in certain tumours (including hepatocellular, prostate, and renal cell carcinoma) and during trophoblast invasion^32–35^, was found significantly downregulated (P < 10^−15^; Fig. 2g and **Extended Data** Fig. 2e). Furthermore, shear stress-stimulated cells displayed an early upregulation of transcription of genes related to reactive oxygen species (ROS) and peroxisome involved oxidative reactions (**Extended Data** Fig. 2f). Vascular endothelial cells produce ROS as an early homeostatic response to shear by regulating their xanthine oxidase activity, nitric oxide release, and protein turnover^36,37^. ROS production has been previously connected to shear stress mechanotransduction by G-protein activation and lead to NF-κB and AP-1 activation via ROS second messenger activity suggesting a possible mechanism for FSS mechanotransduction in HGSC^38^. Overall, the early response to FSS indicates the induction of novel mechanisms involving transcriptional modulation of MUC15 and induction of ROS.

Next, to probe driving molecular pathways after cellular adaptation to flow (24 hr), we performed bulk RNA-seq. Here, not only did steady exposure to 11 dynes/cm^2^ FSS continue to upregulate ROS pathways, but EMT, endoplasmic reticulum (ER) stress, and NF-κB were also found to be significantly activated (Fig. 2h,i). Genes relating to cytoskeleton maturation, including *TAGLN, PARVA, ZYX, LANCL2*, and *ACTA2* increased in expression, while MUC15 was significantly downregulated (Fig. 2h and **Extended Data** Fig. 2g,h). The progression from BrU-seq to RNA-seq implicates EMT pathways and MUC15 downregulation as potential effectors modulating mechanotransduction. Given the extensively altered molecular landscape of sheared HGSC, we extended our approach to include the cell of origin, fallopian tube epithelial cells (FTE) represented by the FT237 cell line, to determine if these changes were unique to tumour cells (Fig. 2j). After 24 hr exposure to 11 dynes/cm^2^ FSS, only 106 upregulated genes were shared between OVCAR3 and FT237. Uniquely upregulated genes in OVCAR3 (449 in total) spanned from those associated with tissue development, cellular movement, and cytoskeleton contraction, to IL-1 and MAPK pathways (Fig. 2k,l,m and **Extended Data** Fig. 2i,j). These results imply ascitic FSS mediates a unique phenotype in HGSC that spans EMT and involves cytoskeleton maturation, NF-κB, and MAPK.

### MUC15 as a biomarker for ascitic shear stress mediated EMT

**Figure 3:**
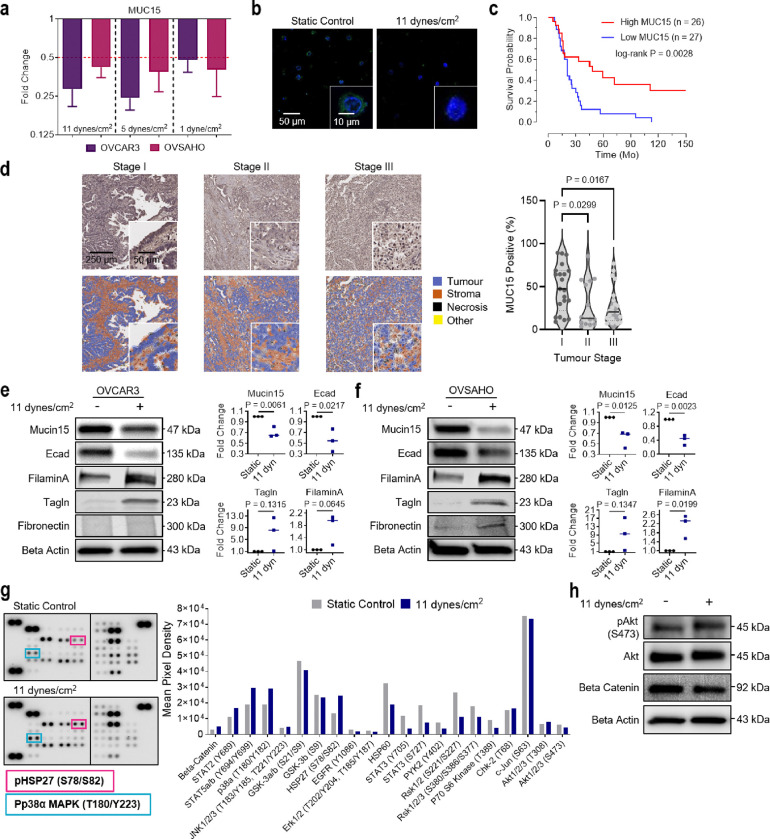
FSS induces a mesenchymal-like phenotype and downregulates MUC15 in ovarian cancer cells. **a,** Fold change of MUC15 mRNA expression in sheared OVCAR3 and OVSAHO cells compared to static controls (1 dynes/cm2, 5 dynes/cm^2^, 11 dynes/cm^2^; 24 hr; n = 3). **b**, MUC15 immunofluorescence of recovered OVCAR3 from static and sheared conditions (11 dynes/cm^2^, 24 hr). **c**, Kaplan-Meier curve showing overall survival in late-stage, high-grade papillary serous ovarian cancer patient cohort with either low or high MUC15 mRNA expression. Significance evaluated by Mantel-Cox test. **d**, MUC15 immunohistochemistry and quantification of MUC15 fold change between stage I, II, and III HGSC patients. Significance evaluated by one-way ANOVA, multiple comparisons. **e,f**, Protein expression and quantification of MUC15 and EMT markers in static and sheared OVCAR3 (**e**) and OVSAHO (**f**) cells (11 dynes/cm^2^, 24 hr). Significance evaluated by Student’s t-test. **g**, Phospho-kinase array and the associated quantification of suspected signal transducers in static and sheared OVCAR3 cells (11 dynes/cm^2^, 24 hr). **h**, Protein expression of suspected FSS-associated signaling molecules in static and sheared OVCAR3 (11 dynes/cm^2^, 24 hr).

As significant loss of *MUC15* expression was observed at both the early onset and after long-term exposure (24 hr) to elevated FSS, we performed a systematic analysis at variable shear conditions to determine if this trend was conserved across different stress levels and cell lines. OVCAR3 and OVSAHO cells exposed to 1, 5, and 11 dynes/cm^2^ for 24 hr displayed consistent downregulation of *MUC15* (Fig. 3a,b). Moreover, low *MUC15* expression was closely associated with poor overall survival in patients (P = 0.0028; Fig. 3c)^39^. In patient-derived tumour samples, MUC15 expression was observed to decrease from stage I to stage II (0.60-fold change; P = 0.0299) and stage III (0.56-fold change; P = 0.0167; Fig. 3d). However, no change in MUC15 expression was observed between primary tumours (stage III and IV) and associated metastatic sites (0.96-fold change; P = 0.1294; **Extended Data** Fig. 3a). Since the application of ascitic FSS enhanced migration and invasion in HGSC cells, we hypothesised that ascitic flow promotes EMT. We probed HGSC cells using a panel of antibodies against proteins including, MUC15, E-cadherin, filamin A, transgelin, fibronectin, vimentin, and N-cadherin.

Sheared HGSC cells (OVCAR3 and OVSAHO) displayed significantly elevated filamin A (1.73 and 2.13-fold change), transgelin (7.26 and 9.52-fold change), and a decrease in E-cadherin (0.55 and 0.42-fold change) consistent with EMT, however, there were no significant differences in fibronectin, vimentin, or N-cadherin (Fig. 3e,f and **Extended Data** Fig. 3b). This EMT-like state has been described previously, outlining epithelial and mesenchymal hybrid stages in ovarian cancers^40^. These hybrid states promote cell survival and aid in dissemination, anoikis resistance, and mesothelial clearance^41^. Previous work demonstrates that FSS mechanotransduction at FSS less than 1 dyne/cm^2^ acts through PI3K/Akt pathways to promote stemness and chemoresistance in ovarian cancer^9,42^. However, ascitic levels of FSS (11 dyne/cm^2^) instead activate p38α MAPK and downstream effector heat shock protein 27 (HSP27; Fig. 3g,h and **Extended Data** Fig. 3c).

### Shear stress regulates p38α MAPK and NF-κB signaling

**Figure 4:**
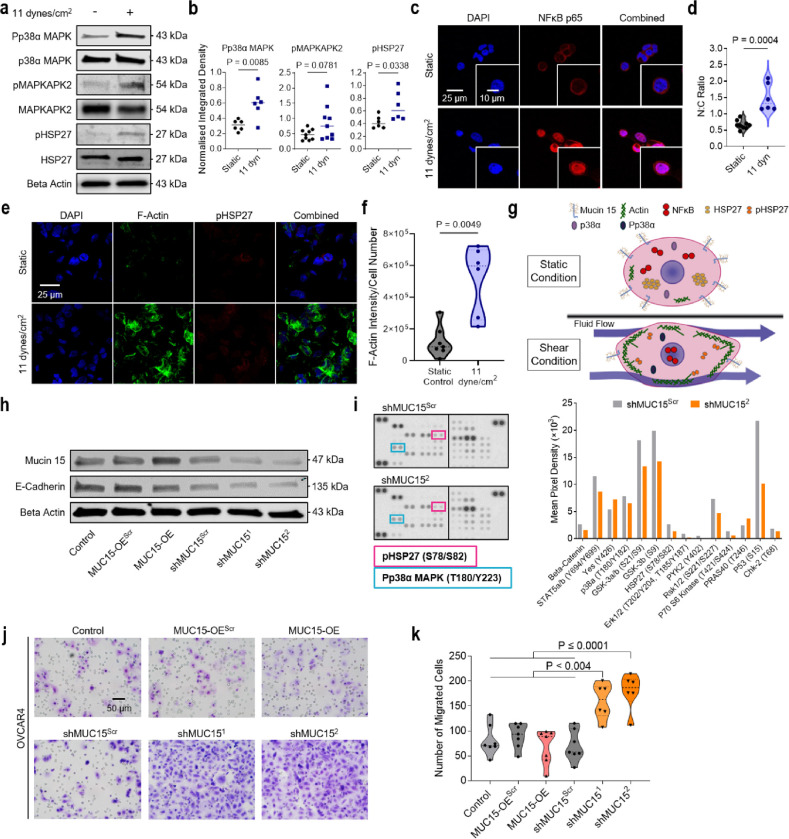
FSS activates p38α MAPK and NF-κB signaling. **a,b,** OVCAR3 were exposed to static or 11 dynes/cm^2^ FSS conditions for 24 hr. Protein expression (**a**) and quantification (**b**) of p38α MAPK-associated signaling molecules. Significance evaluated by Student’s t test. **c,d**, NF-κB p65 immunofluorescence of recovered OVCAR3 (**c**) and quantification of the nuclear-to-cytoplasmic (N:C) ratio of NF-κB signal (d). Significance evaluated by Student’s t-test. **e,f,** F-actin and pHSP27 immunofluorescence (**e**) and the associated quantification of F-actin intensity (**f**). Significance evaluated by Student’s t-test. g, 24 hr FSS stimulation results in a downregulation of MUC15, activation of p38α MAPK, HSP27, and NF-κB signaling, and increased cytoskeletal remodeling. **h**, Protein expression of the OVCAR4 parental, MUC15-overexpressing vector control (MUC15-OE^Scr^), MUC15-overexpressing OVCAR4 (MUC15-OE), MUC15 shRNA control (shMUC15^Scr^), and MUC15 knockdown OVCAR4 (shMUC15^1^/shMUC15^2^ ) cell lines. **i**, Phopsho-kinase array and quantification of suspected FSS signal transducers in MUC15 knockdown and scrambled shRNA control OVCAR4**. j,k**, Boyden chamber migration assay (**j**) and quantification of the number of migrated cells per FOV (**k**) of MUC15-transduced OVCAR4. Significance evaluated by one-way ANOVA, multiple comparisons.

In vasculature, activation of p38α MAPK and downstream effector HSP27 has been shown to aid in the process of endothelial cell alignment and actin filament remodeling in response to FSS^43^. Here we also found active phospho-p38α MAPK and phsopho-HSP27 increased 1.94-fold (P = 0.0085) and 1.60-fold (P = 0.0338) respectively under FSS (Fig. 4a,b and **Extended Data** Fig. 4a). FSS also induced significant nuclear translocation (2-fold increase) of NF-κB in OVCAR3 (Fig. 4c,d) which is known to be elevated in malignant and borderline serous ovarian tumours^44^. The p38α MAPK and NF-κB pathways, which are promising targets for therapeutic intervention, are also critical in the processes of ovarian cancer transdifferentiation and survival^45,46^. Actin monomers are shuttled by the molecular chaperone dimer, HSP27, promoting cell migration and cytoskeleton maturation as well as metastasis in ovarian carcinomas^47,48^. Sheared cells developed robust cortical actin structures consistent with HSP27 dimerization and increased shuttling (Fig. 4e,f). Altogether, stimulated HGSC cells display activated NF-κB, HSP27, and p38α MAPK signaling, as well as a resounding cytoskeletal reorganisation (Fig. 4g).

As MUC15 dysregulation is context-dependent in disease (e.g. lower expression in prostate and hepatocellular metastatic cancers, and is overexpressed in renal carcinomas and preeclampsia), we sought to determine if MUC15 downregulation was sufficient to activate both p38α MAPK signaling and enhanced migration in HGSC. We produced lentiviral knockdown (shMUC15) and overexpressed (MUC15-OE) MUC15 cell lines on OVCAR3 and OVCAR4 backgrounds (Fig. 4h and **Extended Data** Fig. 4b). Although shMUC15 enhanced cellular migration (P ≤ 0.004 and P ≤ 0.001 for shMUC15^1^ and shMUC15^2^ respectively), it was not directly through p38α MAPK signaling (Fig. 4i–k and Extended Fig. 4c-i). Proliferation in shMUC15 lines was also unaffected, whereas MUC15-OE displayed a 0.36-fold decrease in cell division (**Extended Data** Fig. 4j-m). Therefore, by utilising shMUC15 and MUC15-OE, HGSC cell lines we found that although MUC15 was not sufficient to activate p38α MAPK, MUC15 downregulation does induce cellular migration.

### Ovarian cancer senses FSS through GPCRs and MUC15

**Figure 5:**
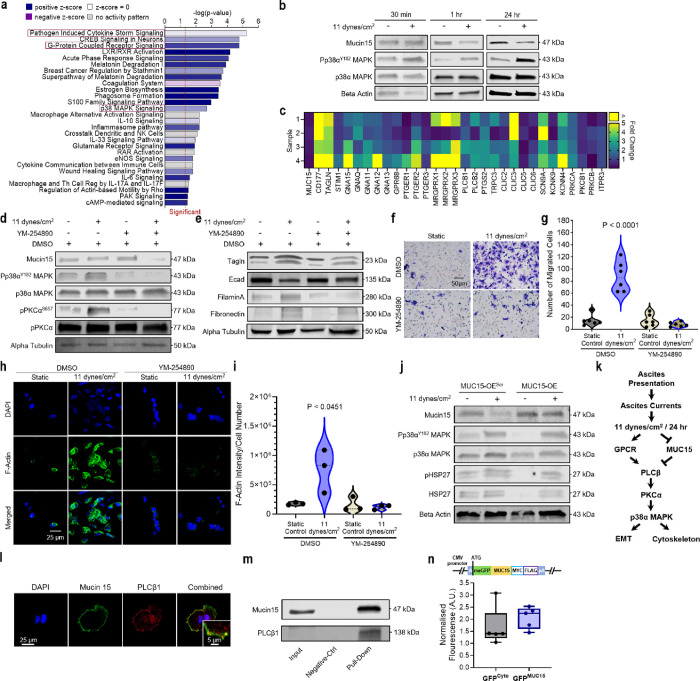
GPCRs and MUC15 mediate FSS mechanotransduction in ovarian cancer cells. **a**, Pathway analysis based on RNA-seq of static and sheared OVCAR3 (11 dynes/cm^2^, 24 hr). b, Protein expression of OVCAR3 cells exposed to static or 11 dynes/cm^2^ FSS conditions for 30 min, 1 hr, or 24 hr. c, mRNA heat map showing the expression fold change of suspected mechanosensitive signaling molecules for pairs of static and sheared OVCAR3 (11 dynes/cm^2^, 1 hr). **d,e**, Protein expression of MUC15 and p38α MAPK-associated signaling molecules (**d**) and EMT markers (**e**) in static and sheared OVCAR3 (11 dynes/cm^2^, 24 hr) with or without YM-254890, a Gα_q_ inhibitor. **f,g**, Boyden chamber migration assays (**f**) and quantification of the number of migrated cells per FOV (**g**) of static and sheared OVCAR3 (11 dynes/cm^2^, 24 hr) with or without YM-254890. Significance evaluated by one-way ANOVA, multiple comparisons. **h,i** F-actin immunofluorescence (**h**) and quantification F-actin intensity (**i**) of OVCAR3 (11 dynes/cm^2^, 24 hr) with or without YM-254890. Significance evaluated by one-way ANOVA, multiple comparisons. **j**, Protein expression of static or sheared MUC15-OE^Scr^ and MUC15-OE OVCAR3 cells (11 dynes/cm^2^, 24 hr). **k**, Schematic of the proposed ascitic FSS mechanotransduction pathway. **I**, MUC15 and PLCβ1 immunofluorescence in OVCAR3. **m**, MUC15 and PLCβ1 co-immunoprecipitation assay in OVCAR3. **n**, N-terminal GFP-MUC15 fusion protein promoter and gene sequence (top). Fluorescent intensity of recovered conditioned medium from static and sheared GFP^Cyto^ and GFP^MUC15^ (11 dynes/cm^2^, 24 hr; bottom). Significance evaluated by Welch’s t test.

Although we established MUC15 as a biomarker for FSS exposure, the mechanism by which ascitic FSS is sensed and transmitted to activate downstream signaling in HGSC remains unclear. As MUC15 knockdown alone was not sufficient to activate p38α MAPK, we performed a predictive pathway analysis (Ingenuity Pathways Analysis; IPA) to ascertain mechanosensors upstream of p38α MAPK (Fig. 5a). Not only was p38α MAPK signaling present in the predictions, but multiple signaling pathways involving GPCRs were anticipated candidates as well. GPCRs, including GPR68 and MAS-related G protein-coupled receptors (MRGPRX), sense blood flow in endothelial cells, releasing IP3 and downstream intracellular calcium to vasodilate and remodel vessels^14^. These signaling cascades have also been shown to activate ER stress via ERE1^49^ and NF-κB and MAPK in intestinal epithelial cells and osteocytes^50,51^. As the earliest time-point for both MUC15 loss and phospho-p38α MAPK activation was after an hour of FSS, we probed genes known to be upstream of MAPK signaling at that time (Fig. 5b,c). These results suggested GPCR acts through the Gαq/PLCβ/PKCα signaling pathway to promote p38α MAPK activation. To test this theory, we treated shear stimulated HGSC with a Gαq inhibitor, YM-254890 (YM)^38,52^. Shear-induced phospho-p38α MAPK and intermediary phospho-PKCα signals were mitigated by the addition of YM (Fig. 5d and **Extended Data** Fig. 5a). Moreover, the robust upregulation of EMT markers (Fig. 3), cellular migration (P ≤ 0.0001; Fig. 2), and actin filament remodeling (P = 0.0169; Fig. 4) observed under FSS were abrogated by inhibition of Gαq (Fig. 5e–i). To compare these changes to the HGSC cell of origin, FT237 were subjected to ascitic FSS for 24 hr. Shear exposed FT237 showed no alteration in MUC15 expression or the activation of p38α MAPK or PKCα (**Extended Data** Fig. 5b,c). Since MUC15 loss alone was not sufficient to promote p38α MAPK in HGSC, we theorised that MUC15 loss was instead necessary for FSS-induced signaling. To evaluate the role of MUC15 in shear sensing, we applied 11 dynes/cm^2^ for 24 hr to MUC15-OE cells and scrambled controls. We then analysed MUC15, phospho-p38α MAPK, and phoshpo-HSP27 protein levels and found overexpressing MUC15 maintained p38α MAPK activation under FSS. However, the basal level of phospho-p38α MAPK and phospho-HSP27 was completely abrogated in the static control (Fig. 5j). These results suggest FSS mediated p38α MAPK mechanotransduction occurs through the GPCR/Gαq/PLCβ/PKCα signaling axis and involves the loss of MUC15.

The cytoplasmic tail of MUC15 contains a pleckstrin homology (PH) binding domain and PDZ binding motifs^53^. As the cytoplasmic protein, PLCβ1, contains a unique PH domain and localizes preferentially at the plasma membrane^54,55^, we hypothesised that MUC15 may inhibit G-protein signaling by direct (PH domain) or indirect (PDZ mediated) molecular complexing with PLCβ1 (Fig. 5k)^56^. Cells in static adherent cultures were fixed and co-stained for MUC15 and either PLCβ1 or PLCβ2. We discovered that PLCβ1 co-localised with MUC15 at the cell membrane, while PLCβ2 instead appeared dispersed throughout the cytoplasm and did not localise with MUC15 (Fig. 5l and **Extended Data** Fig. 5d). Moreover, PLCβ1 was found present in the elution of immunoprecipiate after an overnight incubation with anti-MUC15 conjugated beads (Fig. 5m). This data indicates PLCβ1 and MUC15 may form a molecular complex at the cell membrane. Although MUC15 has no identifiable proteolytic domains, its concentration has been measured in both maternal serum during pregnancy and bovine milk serum^33,57,58^. To test the cleavage potential of MUC15 under FSS, we created a MUC15 N-terminally labeled eGFP fusion protein (GFP^MUC15^; **Extended Data** Fig. 5e) that when cleaved would produce a labeled, extracellular compartment. GFP^MUC15^ cells were then conditioned with FSS and compared to cytoplasmic expressing eGFP controls (GFP^cyto^). Fluorescence was then measured from conditioned medium. We observed no change in fluorescence intensity from normalised GFP^MUC15^ medium or from protein in conditioned medium when compared to controls (Fig. 5n and **Sup. Figure 3**). However, encapsulated GFP^MUC15^ cells lost fluorescence signal when sheared (**Extended Data** Fig. 5f).

### FSS induces an immunomodulatory secretome

**Figure 6:**
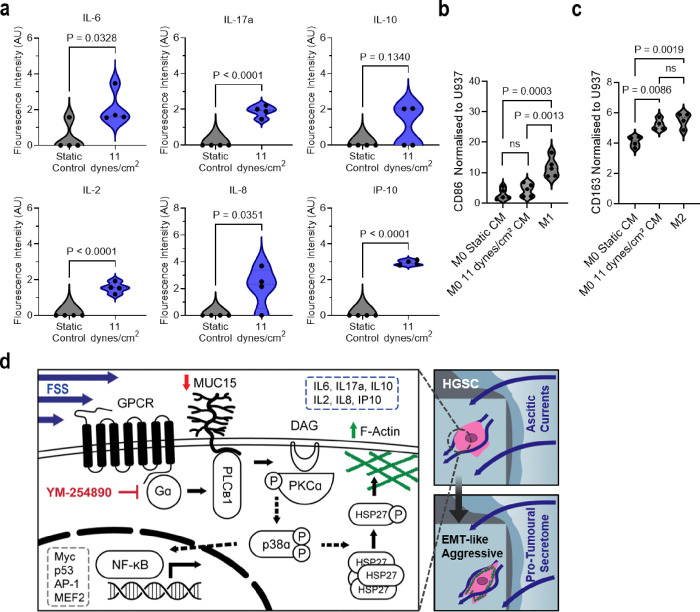
FSS stimulation induces immunosuppressive behavior in ovarian cancer cells. **a,** Fluorescent intensity of cytokines secreted by static and sheared OVCAR3 (11 dynes/cm2, 24 hr) in recovered conditioned media and measured by multiplex cytokine array. Significance evaluated by Student’s t-test. **b,c**, CD86 (**b**) and CD163 (**c**) expression of U937-derived M0 macrophages following 24-hr treatment with conditioned media from static and sheared OVCAR3 (11 dynes/cm^2^, 24 hr). Marker expression was normalised to naïve U937 monocytes and compared to positive control U937-derived macrophages polarised to M1 (CD86; **b**) or M2 (CD163; **c**) by chemical treatment. Significance evaluated by one-way ANOVA, multiple comparisons. **d**, Schematic of ascitic FSS mechanotransduction pathway in HGSC.

The expression of cytokines and chemokines within malignant ascites, including chemokine ligand-2 (CCL-2), colony stimulating factor-1 (CSF-1), interleukin (IL)-6, IL-8, and IL-10, polarise circulating monocytes into tumour associated macrophages (TAM)^1^. Representation of TAMs found in HGSC increases with tumour stage and consists of up to 32% of the cell population within the primary tumour and over 50% of cells within malignant ascites^59,60^. As both p38α and NF-κB activation (Fig. 4a–d) and our IPA predicted the initiation of the tumour *cooling* processes (Fig. 5a), we measured 22 adaptive secretome targets within recovered conditioned medium to assess the connection between shear stress and immunomodulation^61,62^ (Fig. 6a and **Sup. Figure 4a**). FSS-induced the secretion of IL-6 (P = 0.0328), IL-17a (P ≤ 0.0001), IL-2 (P ≤ 0.0001), IL-8 (P = 0.0351), and IP-10 (P ≤ 0.0001), as well as a modest increase to IL-10 (P = 0.13), from conditioned HGSC cells (Fig. 6a and **Sup. Figure 4b**). Motivated by the similarities between our FSS-induced secretome and secreted molecules measured in patient ascites^1,3^, we investigated the impact of the FSS-secretome on macrophage fate. Conditioned medium from either static-control gels or the perfusion bioreactor were added to non-activated (M0) macrophages in hanging-drop plate cultures^63^. We found a significant 1.28-fold increase (P = 0.0086) to the expression of the pro-tumoural marker, CD163, when M0 macrophages were subjected to shear conditioned medium (Fig. 6b). However, no significant difference was observed in the expression of the anti-tumoural marker, CD86 (Fig. 6c). These results demonstrate elevated FSS induces a pro-tumoural secretome that further promotes TAM polarisation and may help explain in vivo molecular characteristics of ascites.

## Discussion

Transcoelomic metastasis substantially contributes to high morbidity rates in HGSC patients^1,4^. However, the initiation and mechanisms that support this process remain obscure. Here we detail the formation of ascitic currents and resultant FSS experienced by the primary tumour. Further, we demonstrate GPCR/MUC15/p38α MAPK mechanosignaling and a shear-induced mesenchymal-like phenotype, clarifying the link between ascites retention and poor patient prognosis^5,6^.

Established models of localised fluid motion corresponding to gastrointestinal peristalsis^19^ neglect the physical circulation of ascites as a source of FSS. Considering the average ascites volume can exceed 2,500 mL, we modeled the effect of subdiaphragmatic pressures on the motion of ascites^64^. CFD methodology previously developed for cardiac hemodynamics involving Newtonian fluids, were utilised in this approach^25,26^. Our findings suggest that ascitic FSS from diaphragmatic contractions lie orders of magnitude higher than previously thought^16,17^. It is expected that a dynamic range of FSS is present when considering a diverse population. Future work will benefit from a larger patient cohort and sampling from both discrete stage (I-IV) and ascites volume. Use of magnetic resonance imaging to monitor cavity deformation over the action of diaphragmatic excursion may also improve model resolution and clarity.

Although FSS is widely accepted to promote metastasis, our findings present a nuanced view on the effects of both long-term and elevated FSS exposure on HGSC progression. Our results confirm the activation of EMT and the NF-κB pathways. However, contrary to previous findings, EMT from ascitic FSS may not be dependent on PI3K/Akt but rather on the activation of p38α MAPK^11,42^. Cells contain abundant mechanosensors, with distinct sensitivity and temporal reactivity that signal collectively to inform cellular decision. Previous reports show various conserved roles of GPCRs in mechanosensing, such as direct shear sensing of GPR68 in small diameter resistant arteries^14^ and the indirect mechano-chemical signaling of cochlear planar cell polarity in auditory development^65^. Our Gα inhibitor studies develop the FSS signaling axis to include GPCR mechanotransduction in addition to PLCβ, PKCα, and downstream MAPK. Furthermore, these results provide new insight into the connection between FSS and observed cytoskeleton maturation in HGSC^19^, suggesting F-actin maturation is mediated via MAPKAPK2 and HSP27. This positions GPCR/p38α MAPK shear signaling as a therapeutic target to reduce peritoneal spread in HGSC patients, potentially through the use of YM-254890.

The dysregulation of MUC15 is observed in many cancers with links to EMT via EGFR inhibition, PI3K-Akt, and GSK3β/β-catenin signaling^32,35^. Our data indicates that HGSC patients with lower MUC15 have poorer overall survival and that MUC15 expression is lost during ovarian cancer progression. Additionally, when secondary peritoneal tumours (stage III and IV) were compared to paired primary sites, MUC15 expression was not found to change. This data suggests the most significant loss of MUC15 occurs within the primary tumour between stages I and II, when ascites accumulation first begins rather than during peritoneal dissemination.

The glycocalyx is integral to the transduction of FSS to the lipid membrane and plays a direct role in mechanosignaling, for instance, the digestion of both hyaluronic acid and heparin sulfate have been shown to reduce shear-induced nitric oxide production in isolated arterioles^66,67^. MUC15 is one of the smallest of the transmembrane mucins and little is known about its activity. Although, MUC15 is downregulated in the process of extravillous trophoblast invasion toward maternal spiral arteries and is also released by lactocytes in mammary glands, a direct link between MUC15 and FSS had not been established^33,53^. Our results show MUC15 is lost in as little as an hour after exposure to FSS. Steric hindrance and hydraulic resistivity from larger mucins may attenuate at higher FSS allowing mucins closer to the membrane surface to transmit shear signal more effectively^10^. This may explain why MUC15 downregulation has not yet been described in FSS in vitro findings as many are performed at low FSS^7^. Sheared conditioned medium and encapsulated cells from eGFP N-terminally labeled MUC15 suggest MUC15 does not release under elevated shear conditions but may initiate cellular recycling or lead to conformational changes on the C-terminus instead. Our findings suggest ascitic FSS signal MUC15 to release PLCβ1 to promote GPCR, p38α MAPK, and NF-κB signaling in HGSC cells (Fig. 6d). However, further evidence is required to establish MUC15 as a *bona fide* mechanosensor. FT237 cells exposed to FSS do not display changes to MUC15 expression or to the activity of p38α MAPK and PKCα. This potentiates differences in glycocalyx composition as an area of interest for contrasting shear sensitivities of HGSC and FTE.

Together, replicating *in vivo* trends, ascitic FSS is sufficient to induce a robust downregulation of MUC15 and the activation of EMT and immunomodulation through the novel ascitic FSS/GPCR/p38α MAPK signaling axis. These findings underscore the urgent need for targeted interventions, such as in situ pumps or frequent paracentesis, to manage ascites accumulation. Given that malignant ascites – present in various cancers including liver, breast, uterine, cervical, and gastrointestinal – facilitates transcoelomic metastasis, unraveling these mechanisms may lead to maintenance therapies that abate diverse disease and metastasis more broadly.

## Methods

### Cell culturing and live cell recovery.

HGSC representative cell lines OVCAR3 (malignant ascites), OVSAHO (abdominal metastasis), and OVCAR4 (malignant ascites) were purchased from the American Type Culture Collection (ATCC) and cultured in Roswell Park Memorial Institute medium 1640 (RPMI) (Thermo Fisher Scientific; 11875119) with 10% fetal bovine serum (FBS; Gemini Bio-Products; 100–106) and 1% antibiotic/antimycotic (Thermo Fisher Scientific; 11875119). FTE representative cell line FT237 were cultured in Dulbecco’s Modified Eagle Medium/Nutrient Mixture F-12 (DMEM/F12; Thermo Fisher Scientific; 11320033) with 10% FBS and 1% antibiotic/antimycotic. All cell lines were cultured in a humidified atmosphere with 5% CO_2_ at 37°C and were free of mycoplasma contamination (via routine STR profiling and mycoplasma testing). Cells were collected from plates using 0.25% trypsin and pelleted before suspension at 10 million cells/750 μL of 3% (w/v) low gelling temperature agarose (Boston Bioproducts Inc.; P73050G) and 1 mg/mL collagen type I (Cultrex; 344310001), as described previously^27^. Cells were recovered from encapsulation using our previously outlined methodology^27^, briefly 150 units of agarase from *Pseudomonas atlantica* (Sigma; A6306) were added to finely minced hydrogels suspended in 750 μL of RPMI. The cell plus hydrogel slurry was then mixed and stirred at 5 min intervals for 45 min before they were resuspended in 10 mL of RPMI, were passed through a 100 μm filter (3×) and then were finally plated on 150 mm tissue culture dishes. After 2.5 hr, the remaining hydrogel chunks were washed off with 1×PBS and cells were trypsinised, counted, and prepared for downstream assays.

### Ascitic Model.

To create CFD models of the ascites under regular and active conditions, CT images were collected from an ovarian patient with moderate to severe ascites and the fluid volume was segmented (**Extended Data** Fig. 1a, left). From the segmentation, a tetrahedral volumetric mesh was generated in Simmodeler with boundary layers. Isovolumetric deformation was applied so that the region at and below the ovaries was held stationary while the top was modeled to deform vertically, driven by the diaphragm (**Extended Data** Fig. 1a, right). Volume preservation was ensured by introducing equal lateral deformations. Deformation decreased linearly from the top of the ascites (maximum deformation) to the ovaries (no deformation). For regular breathing, the maximum deformation was 6.0 cm with a frequency of 0.37 Hz. For active conditions, the maximum deformation was 8.0 cm with a breathing frequency of 1.0 Hz.

This prescribed deformation was used to calculate domain wall velocity, which was propagated through the 3D mesh using a linear elastic problem^25^. The solution was then incorporated into the Arbitrary Lagrangian-Eulerian (ALE) Navier-Stokes Eq. 6^8^. To predict the flow (pressure and velocity), we utilised the finite-element based solver Cheart to perform CFD simulations^68^. Ascites fluid flow was modeled as an incompressible, Newtonian fluid with a density^69^ of 1017.5 kg/m^3^ and a viscosity^70^ of 0.0012 Pa·s. A no-slip condition was applied to the walls of the ascites and ovaries. The problem was run for three diaphragmatic cycles to achieve a steady state. The resulting velocity field was used to calculate the wall shear stress^71^.

### Live cell-collagen fluorescent confocal imaging.

Live OVCAR3 cells and collagen fibers were simultaneously imaged by fluorescent and second harmonic generation (SHG) confocal microscopy. GFP-labelled OVCAR3 cells were suspended within agarose-collagen type I IPN hydrogels at 13.33 million cells/mL and imaged with a Leica SPX8 laser scanning multiphoton confocal microscope. SHG imaging was performed using an excitation wavelength of 860 nm and a collection window of 425 to 435 nm. A 40× water emersion lens with a 100 Hz scan speed was utilised to capture z-stack images. EGFP labelled OVCAR3 cells were imaged in situ after an hour of encapsulation.

### Perfusion bioreactor construction and use.

Bioreactors were machined from 316 stainless steel for its bio-inertness, autoclave compatibility, and resistance to corrosion. The reactors first designed in SolidWorks^™^, were milled at the University of Michigan Department of Physics, Scientific Instrument Shop (**Extended Data** Fig. 1c,d). Shear bioreactor construction, assembly, and use have been described elsewhere^28^, the present version contains the addition of flow entry and exit compartments surrounding hydrogel stacks to improve flow homogeneity and press-fit rods to ease sample retrieval (**Extended Data** Fig. 1d). Briefly, continuous cell culture medium was pumped through the reactor from the medium reservoir, containing a volume of 20 mL of cell culture medium per cell containing hydrogel (**Extended Data** Fig. 1b). Experiments were performed for either 0.5, 1, or 24 hr with continuous applied pulsatile flow. For runs using YM, the compound was dissolved in the perfusate to final concentrations of 100 nM. YM inhibitor experiments were performed for 24 hr. Each experimental condition was repeated 3–6 times.

### Computation modelling of shear stress.

Computational analysis of bioreactor-related FSS have been developed previously^28^. Briefly, material properties were determined through direct measurement of the hydrogels via rheometry and mercury porosimetry^27^. COMSOL Multiphysics 5.5 was utilised to represent the three-dimensional bioreactor and evaluate shear stresses on an idealised cell (Fig. 1e,h,i). A mesh convergence study was conducted to confirm accuracy of shear values within the bioreactor (**Extended Data** Fig. 1e).

### Shape factor.

Stimulated cell laden hydrogels (1, 5, and 11 dynes/cm^2^), or static control conditions were placed in 10% formalin, paraffin embedded, and sectioned (5 μm; perpendicular to the flow direction). Slides were then stained for hematoxylin and eosin (H&E) and imaged under a light microscope at 40× magnification for morphometry and shape factor analysis. These images were analysed using NIH ImageJ to quantify cellular area, aspect ratio, perimeter, roundness, and circularity. A minimum of three biological replicates were analysed, each containing eight technical replicates with at least 160 cells per condition.

### Migration and invasion assays.

Migration and invasion assays were performed using Boyden chambers (8 μm pores; Transwell; Corning Incorporated). For invasion assays, Matrigel^™^ (ECM Gel from Engelbreth-Holm-Swarm murine sarcoma, Sigma; E6909) was reconstituted to 5 mg/mL in serum-free RPMI 1640, and 40 μL was allowed to gel in the upper chamber for 2 hr at 37°C. For both migration and invasion assays, cells were resuspended in serum-free RPMI 1640 and added to the upper chamber. For shear OVCAR3/OVSAHO studies, 100,000 and 150,000 cells were plated for migration and invasion assays, respectively. For MUC15-transduced OVCAR3/4 studies, 50,000 and 100,000 cells were plated for migration and invasion assays, respectively. The inserts were placed within the wells of a 24-well plate containing RPMI 1640 supplemented with 10% FBS. After 24 hr in an incubator, medium from the upper chamber was discarded and Matrigel^™^ was cleaned from the Boyden chamber membrane. Cells were fixed in 10% formalin for 10 min, permeabilised in methanol for 20 min, and dyed with 0.2% (w/v) crystal violet for 20 min. Cells that had not migrated or invaded were cleaned from the top face of the membrane, and the membranes were allowed to dry. Membranes were mounted with toluene mounting solution (Fisher Scientific; SP15–100). Bright field imaging was performed with an Olympus IX83 fluorescent microscope (20× long working distance). Six representative images were taken for each sample. Migrated or invaded cells were counted for each field of view and averaged over the image.

### Bru-pulse and bulk-RNA sequencing.

For nascent RNA experiments, 5-bromouridine was injected into the medium reservoir to a final concentration of 2 mM (at 30 min; 11 dynes/cm^2^; OVSAHO; n = 2) and static control plates for a 30 min bru-labelling pulse. Cells were left to be exposed to FSS or static conditions and incubated at 37°C, afterwards cell laden hydrogels were recovered, washed in 1×PBS (3×), and lysed in buffer RLT RNA extraction reagent (1 mL per sample; Qiagen) with 1% β-mercatol ethanol to collect total RNA (> 100 μg). Bru-labelled RNA was isolated from the total RNA by incubation with anti-BrdU antibodies (BD Biosciences) conjugated to magnetic beads (Dynabeads, Goat anti-Mouse IgG; Invitrogen) under gentle agitation at room temperature for 1 hr. Isolated bru-labelled RNA was used to prepare strand-specific DNA libraries by using the Illumina TruSeq Kit (Illumina) according to the manufacturer’s instructions with modifications shown elsewhere^72^. For bulk-RNA, OVCAR3 or FT237 laden hydrogels were recovered from shear and static conditions (11 dynes/cm^2^; n = 3) at 24 hr and lysed in buffer RLT to collect total RNA (RNeasy Mini kit; Qiagen). Sequencing of the cDNA libraries prepared from nascent and total RNA was performed at the University of Michigan Sequencing Core by using the Illumina HiSeq. 2000 sequencer. Gene-set enrichment analysis (GSEA) and over-representation analysis (ORA) using the hallmark pathway were performed on protein coding differential gene expression from Bru-seq and bulk-RNA seq data with a p-adjusted value of at least 0.01 and a transcripts per million cut off equal to 1 and a log 2 fold change cut-off for GSEA and ORA respectfully. Ingenuity pathway analysis (IPA) was performed utilising Qiagen proprietary IPA software.

### Real-time quantitative PCR.

Total RNA was isolated from all 1 and 24 hr shear stressed treated HGSC cell lines using buffer RLT RNA extraction reagent (1 mL per sample) with 1% β-mercatol ethanol on minced hydrogels (overnight at 4°C or until the hydrogel has fully dissolved) and then cDNA was synthesised using High Capacity cDNA Reverse Transcription Kit (Applied Biosystems). The quantitative PCR was performed by using PowerUP SYBR Green Master Mix (Applied Biosystems) with gene-specific primers in triplicate according to the manufacturer’s instructions. The primer sequences are listed in **Supplementary Table 1**. Relative fold expression for genes of interest were calculated using the comparative CT method with *tubulin (TBP), hypoxanthine phosphoribosyl transferase 1 (HPRT1), Ribosomal Protein L27 (RPL27)* as the internal controls, *CD177* and *transgelin (TAGLN)* as positive control. Expression levels of mechanical sensor markers in HGSC cells were normalised to internal controls.

### Kaplan-Meier survival analysis.

A cohort of advanced ovarian cancer patients (N = 53) from Mok et al. were classified based on *MUC15* expression^39^. This data was identified using the cBioPortal application, GSE18521. The average patient age was 61.9 years (SD = 12.7), with an average survival time of 40.5 months following surgery (SD = 41.3 months). Micro-dissected distinct populations of epithelial cells were sequenced utilising a whole-genome Affymetrix U133 Plus 2.0 GeneChip microarray. Kaplan-Meier survival analysis was generated using GraphPad Prism, v.5.0 (GraphPad Software).

### Immunoblotting.

Western blotting was performed according to standard procedures. Hydrogels were recovered, minced, washed with 1×PBS, and then lysed using 550 μL RIPA with 2× Halt^™^ (Thermo Scientific) and 2 mM phenylmethylsulfonyl fluoride (PMSF; Roche; 30 min; 4°C). The supernatant was then processed through sonication and spun to remove cellular debris (30 min; 4°C; 17,000 g). Samples were standardised, boiled (5 min), prepared with 1× Laemmli buffer, loaded into 4–15% Mini-Protean TGX gels (BioRad), and subsequently transferred to a polyvinylidene difluoride (PVDF) membrane using wet transfer. The PVDF membranes were blocked in 5% bovine serum albumin (BSA) in Tris-buffered saline with 0.1% Tween20 (TBST) for 45 min and probed with primary antibodies diluted in 3% BSA in TBST overnight at 4°C and subsequently with secondary antibody conjugated to DyLight^™^ 800 4x PEG (Invitrogen) in 5% milk in TBST (90 min; room temperature). Protein bands were detected using an Odyssey DLx (LiCore) and were quantified in NIH ImageJ. A list of all the antibodies used in this study, including their working dilutions, can be found in **Supplementary Table 2.**

### Tissue microarray immunohistochemistry.

Tissue microarrays containing 40 pairs of primary and metastatic ovarian tumours and also stage I-IV ovarian tumours were purchased from TissueArray. Slides were baked for 60 min at 60°C before deparaffinising and rehydrating. Heat-induced epitope retrieval was performed for 30 min in Tris-EDTA Antigen Retrieval Buffer (Proteintech; PR30002) supplemented with 0.05% Tween20. Endogenous peroxidase activity was blocked with BLOXALL^®^ Blocking Solution (Vector Laboratories, Inc.; SP-6000). Samples were then blocked for 2 hr in 10% Goat Serum, 2% BSA, 22.5 mg/mL glycine, 0.1% Tween20 in 1× PBS before overnight treatment with a MUC15 primary antibody (Sigma-Aldrich; HPA026110; 1:20) at 4°C. Samples were incubated with an HRP-secondary antibody (Bio-Rad; 5196 − 2504; 1:400) for 90 min at room temperature. MUC15 staining was performed with a DAB chromogen substrate (Thermo Scientific; 34002). Finally, samples were counterstained with hematoxylin (Poly Scientific R&D Corp.; s212A-32OZ), dehydrated, and mounted with toluene mounting solution (Fisher Scientific; SP15–100). MUC15-expressing tumour cells were quantified with QuPath v0.5.1. The software was trained to identify epithelial-like tumour, mesenchymal-like tumour, stromal, necrotic, and other regions. Hematoxylin stained cells were then evaluated based on DAB stain intensity.

### Antibody arrays.

For antibody arrays, cell lysates were diluted and incubated overnight (4°C) with the human Phospho-Kinase Array (Proteome Profiler; R&D Systems; ARY003C) following the manufacturer’s instructions. Densitometry values were measured via NIH ImageJ and expressed as arbitrary units. The average signal of each duplicate pair of protein, was calculated after the subtraction of the background intensity (pixel density) from negative controls and normalisation to average positive control values.

### ELISA measurements.

Conditioned medium and cell lysates were snap frozen in liquid nitrogen. GFP and MUC15 concentrations were measured by the University of Michigan Immunology Core using GFP (Abcam; ab171581) and MUC15 (Cusabio; CSB-EL015218HU and Abbexa; abx250464) ELISA kits as per the manufacturer’s protocol.

### Genetic knockdown and ectopic expression.

Lentiviral transduction was used to knock-down MUC15 in OVCAR3 and OVCAR4 cell lines. Two short hairpin mRNA plasmids for MUC15 containing a pGipz vector element, an hCMV promoter, tGFP, and Puromycin resistance were purchased from Horizon Discovery (Accession #NM_01135091; Clone ID #V2LHS_246833 and #V2LHS_69631). A MUC15 plasmid containing pCMV6 vector element, an hCMV promoter, and neomycin resistance was purchased from OriGene (Accession #NM_145650; RC205071) and was made lentiviral ready with Myc-DDK and tGPF tags by the University of Michigan Vector Core. One Shot Stbl3 chemically competent Escherichia coli were transformed with the shMUC15 and MUC15-OE lenti-plasmids and grown in LB with carbenicillin (100 μg /mL) and zeocin (25 μg/mL) or kanamycin (25 μg/mL) for selection pressure and expansion respectively. Human embryonic kidney 293T cells were co-transfected with packaging plasmids (Envelope, GAG PoL, REV, etc.) and the lenti-MUC15 plasmids. After a 48 and 72 hr incubation, viral batches were collected and combined. Transduced OVCAR3/4 cells were sorted via 10 μg/mL puromycin or 10 mg/mL neomycin, respectively for shMUC15 and MUC15-OE for 3 days per week. Flow sorting for GFP expressing cells was also performed monthly. N-terminally eGFP labeled MUC15 vectors (**Extended Data** Fig. 5e) were fabricated and made lentiviral ready by the University of Michigan Vector Core. Transduced MUC15^GFP^ labeled OVCAR3 cells were sorted via 10 μg/mL puromycin for 3 days per week.

### Cytoskeleton immunostaining.

OVCAR3 laden agarose-collagen IPNs, were snap frozen, and prepared for cryosectioning after shear or static treatment. Slides were stored at −80°C prior to staining. Slides were thawed for 35 min at room temperature and subsequently rehydrated for 12 min in 1× PBS. Sections were fixed in 10% formalin for 6 min and incubated with 0.4% Triton X-100 in 1× PBS for 30 min. Blocking was performed for 90 min in StartingBlock^™^ (TBS) Blocking Buffer (Thermo Scientific; YA357048) supplemented with 0.1% Tween20. Hydrogel sections were then incubated with phospho-HSP27 primary antibody (Cell Signalling Technologies; 2401S; 1:100) overnight at 4°C followed by a 90-min incubation with an AlexaFluor647-conjugated secondary antibody (Invitrogen; A27040; 1:200). Finally, F-actin was stained with an AlexaFluor488-phalloidin (Invitrogen; A12379; 1:200) before mounting on slides in SlowFade^™^ Diamond Antifade Mountant with DAPI (Invitrogen; S36964). Imaging was performed with a Nikon A1R HD confocal microscope, with 0.5 μm slices over 12 μm stacks. F-actin signal was quantified using integrated density retrieved in NIH ImageJ that was normalised to the cell number per image.

### 2D adherent cell immunostaining.

O OVCAR3 cells were plated on 4-well Millicell slides at a density of 25,000 cells per well. Cells were fixed in 10% formalin for 9 min and permeabilised for 5 min in 0.05% Triton X-100 in 1× PBS. Cells were blocked for 2 hr in 10% Goat Serum, 2% BSA, 22.5 mg/mL glycine, 0.1% Tween20 in 1× PBS before incubating in AlexaFluor488-MUC15 (Santa Cruz Biotechnology, sc-365746 AF488, 1:50) and PLCβ1 (Invitrogen, PA5–76089, 1:50) or PLCβ2 (MyBioSource.com, MBS9606175, 1:400) or AlexaFluor594-NFκB p65 (Santa Cruz Biotechnology; sc-8008 AF594; 1:100) primary antibodies overnight at 4°C. An AlexaFluor647-secondary antibody (Invitrogen, A27040, 1:500) was added for 90 min before mounting in SlowFade^™^ Diamond Antifade Mountant with DAPI (Invitrogen; S36964). Samples were imaged with a Nikon A1SI laser scanning confocal microscope.

### Gα Inhibitor Toxicity Crystal-Violet Assay.

3000 OVCAR3 cells per well were seeded in 96 wells plate and then treated with Gαq inhibitor, YM-254890 (0.01 nM, 0.1 nM, 1 nM, 10 nM, 100 nM, 1000 nM 10000 nM), for 24 hr. The cells were fixed with 10% formalin and incubated with 0.5% crystal-violet (crystal-violet 7·5 g/l, NaCl 2·5 g/l, formaldehyde 1·57%, methanol 50%) for 30 min at room temperature on the bench rocker. To measure the viability of OVCAR3 cells, the crystal-violet solution is removed. Cells are carefully washed 5 times and allowed to be air dried before measurement by an automated microplate reader at Optical Density 593 nm (Biotek Synergy HT Microplate Reader).

### Co-immunoprecipitation.

OVCAR3 were plated on 10-cm plates and lysed at 70% confluency. Lysates and co-immunoprecipitation was performed according to manufacturer’s instructions (Thermo Scientific; Pierce^™^ Co-Immunoprecipitation Kit; 26149). Pierce agarose resin was coupled to anti-MUC15 (100 μL; Sigma) and incubated with lysate overnight at 4°C on a rocker. The beads were collected and the protein was eluted for analysis via SDS-PAGE and protein detection with different antibodies. The antibodies used for the immunoprecipitation are described in **Supplementary Table 2.**

### Multiplex cytokine array.

Media was collected 24 hr after seeding from cells in FSS and static conditions. Media was centrifuged, and supernatant was snap-frozen in liquid nitrogen. Samples were thawed on ice, filtered through 0.2 μm filters and 25 μL of each condition was quantified by CodePlex Secretome Adaptive Immune-L Chips-1 (Bruker; CODEPLEX-21.01–1) using an ELISA based multiplex immunoassay for GM-CSF, Granzyme B, IFN-g, IL-13, IL-15, IL-2, IL-4, IL-5, IL-6, IL-7, IL-8, IL-9, IL-17a, IP-10, MCP-1, MIP-1a, MIP-1b, Perforin, sCD137, TNF-α, TNF-β.

### Macrophage polarisation.

U937 cells were cultured in suspension in RPMI with 10% heat-inactivated fetal calf serum (Atlanta Biologics) and 1% antibiotics-antimycotics. U937 cells were harvested and suspended in 5 ng/mL of phorbol myristate acetate (PMA; Sigma Aldrich). This suspension was plated at 20 μL per well in a hanging drop array plate to have a final concentration of 3000 cells/well for differentiation into macrophages. After 48 hr, wells were left untreated for M0 macrophages, treated with 20 ng/mL recombinant human M-CSF (Fisher Scientific) and 20 ng/mL IL-4 (Fisher Scientific) for pro-tumoural macrophage polarization controls, or 100 ng/mL LPS (Sigma-Aldrich) and 20 ng/mL IFN-γ (Peprotech Inc.) for anti-tumoural polarisation controls. M0 macrophages were treated with 2 μL of conditioned medium from FSS or static control conditions. Macrophages were collected after 24 hr for analysis.

### Flow cytometry.

Flow cytometry analysis was performed following our protocols published previously^73^. CD163-APC (Becton Dickinson) antibody along with its associated APC-isotype control was used to identify pro-tumoural macrophages. CD86-APC (Fisher Scientific) antibody and its associated APC-isotype control was used to identify anti-tumoural macrophages^63^. The respective isotype controls were used for conjugated antibodies to set gates for CD86 and CD163. Flow cytometry was performed on the Attune acoustic focusing flow cytometer (Applied Biosystems). At least 15,000 cells were counted for each flow analysis.

### Image analysis.

Quantification of western blots and immunofluorescence images was performed using NIH ImageJ. At least 6 images were taken for each condition (for immunofluorescence), from at least three independent biological repeats (both western blots and immunofluorescence).

### Statistical analysis.

Data are presented as the mean with SD for the indicated number of independently performed experiments (at least n = 3) and were analysed by Student’s or Welch’s t-test or one-way ANOVA, Multiple Comparisons. All statistical analyses were calculated, and all graphs generated, using the GraphPad Software.

## Data Availability

The data are available upon reasonable requests made to the corresponding author.
