# Supplementary material for: Ascitic Shear Stress Activates GPCRs and Downregulates Mucin 15 to Promote OvarianCancer Malignancy
Source: Res Sq. 2024 Nov 25:rs.3.rs-5160301. Originally published 2024 Oct 15. Preprint. [Version 2] doi: 10.21203/rs.3.rs-5160301/v2 (PMC11527234; doi:10.21203/rs.3.rs-5160301/v2)
Supplement: Supplement 1 — Supplementary Table 1 | FSS-Mechanosensor RT-qPCR Primer Sequences Supplementary Table 2 | Antibodies and Working Concentrations [file NIHPPRS5160301V2-supplement-1.pdf]

Supplementary Files

This is a list of supplementary files associated with this preprint. Click to download.

- extendeddata.docx
